# Disrupted Candidacy: A Longitudinal Examination of the Constrained Healthcare‐Access Journeys of National Health Service Workers in Scotland Seeking Supports for Long COVID Illness

**DOI:** 10.1111/hex.70050

**Published:** 2024-10-02

**Authors:** Nicholas Norman Adams, Emma MacIver, Flora Douglas, Catriona Kennedy, Diane Skåtun, Virginia Hernandez Santiago, Nicola Torrance, Aileen Grant

**Affiliations:** ^1^ School of Health Robert Gordon University Aberdeen Scotland UK; ^2^ School of Medicine, Medical Sciences and Nutrition University of Aberdeen Aberdeen Scotland UK; ^3^ School of Medicine University of St Andrews St Andrews Scotland UK

**Keywords:** Disrupted Candidacy, healthcare access, healthcare professionals, illness candidacy, Long COVID, longitudinal qualitative study

## Abstract

**Introduction:**

Evidence examines how persons experiencing Long COVID (LC) struggle to secure healthcare for symptoms. However, few studies examine healthcare workers experiencing LC, nor the complex and multiple difficulties faced when seeking and receiving healthcare.

**Methods:**

This study is based on two phases of longitudinally conducted qualitative interviews, 6 months apart, with National Health Service (NHS) workers experiencing LC, from different occupational roles at NHS locales in Scotland (first interviews, *n* = 50; second interviews, *n* = 44).

**Results:**

Multiple factors restricted healthcare access, including worries about pressuring the NHS and concerns over LC being legitimised. When healthcare was sought, workers struggled to secure support, referrals and treatment. The following reasons were included: (1) context: the restrictive pandemic healthcare context; (2) illness climate: low GP knowledge surrounding LC and how this could be treated, trends for ascribing symptoms to other causes and reluctance to diagnose LC; (3) sense‐making of LC: healthcare availability linked to occupational role identity. To visualise and examine healthcare barriers, *candidacy theory* is applied, drawing inferences between healthcare context, illness climate, sense‐making and identities.

**Conclusion:**

NHS workers' complex journeys represent *Disrupted Candidacy*, intersecting challenges across candidacy domains, restricting the seeking and receiving of LC healthcare. Findings provide insights into why NHS workers resisted and withdrew from healthcare‐seeking, and the barriers they faced when attempting to secure LC support. This study presents a pathway for future LC illness research to use a modified candidacy theory framework.

**Patient and Public Contribution:**

This research focuses on amplifying and learning from lived experiences, and *the voices* of NHS workers in Scotland experiencing LC. Interviews represent primary data for this study; thus, participants and their healthcare journeys are centred in this research and all aspects of production, reporting and output. Explicit discussions of stakeholder group involvement are highlighted in the methods section.

## 1 Introduction

The impacts of COVID‐19 (C19) on the National Health Service (NHS) have been devastating, with wider health and psychosocial effects of C19 well established as negative and significant [1, 2, 3, 4]. Long COVID (LC) represents a complex constellation of symptoms following acute C19 infection, unexplained by other illnesses; it persists for longer than 12 weeks [5] and often disrupts multiple aspects of well‐being [5, 6, 7]. Symptoms such as fatigue, breathlessness and ‘brain‐fog’ are debilitating, impacting quality of life [5]. Au et al. [8] explore LC as largely patient‐defined, discussing how a lack of medical legitimacy drove sufferers to seek symptom verification and illness recognition in online spaces. Similar perspectives have been highlighted [7, 9, 10], and related scholarship examines complexities in diagnosing LC, revealing challenges in understanding barriers to diagnosis and treatment [11]. Other studies explore biomedical perspectives, linking markers of C19 and LC and hypothesising mechanisms underpinning the illness [12]. Despite different foci, scholarship collectively reveals challenges in diagnosing LC, reflecting a global healthcare trend of poor illness understanding and these factors constraining LC treatment and healthcare access.

Currently, little is known about how NHS workers seek and access LC healthcare, and how wider social contexts, LC illness climate and workers' professional occupational identities can influence access challenges. Developing this knowledge is critical, and this study responds to this gap.

### 1.1 Candidacy

**Figure 1:**
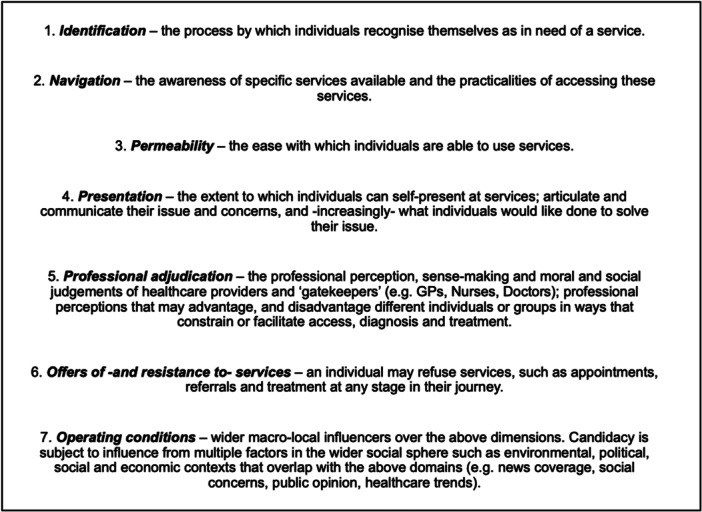
The Candidacy Framework. Candidacy framework, an explanatory interpretation, utilised in some existing works, for example, Mackenzie et al. [15] and Tookey et al. [14].

Candidacy is a well‐established framework for moving beyond simply conceptualising healthcare access barriers as availability and service usability [13, 14]. The Candidacy Framework (TCF) explores how healthcare access is negotiated between individuals and providers [13]. TCF has been applied to understand how and why individuals conceptualise eligibility for healthcare differently. This includes people resisting healthcare, people perceiving themselves as ineligible and undeserving of treatment, and how different factors align to prevent diagnosis and care through socioeconomic, contextual and organisational influencers [14]. Factoring advances in conceptualising illness candidacy [14, 15, 16], an explanatory framework model for candidacy is presented in Figure 1.

Maclean et al. [6] apply TCF to understand LC experiences, interviewing 72 individuals experiencing LC from the United Kingdom, the United States, the Netherlands, Canada and Australia in the first 18 months of the C19 pandemic. Findings highlight ‘vanguard patients’ (p. 1): LC patients collectively identifying, naming and fighting for LC illness recognition, largely via social media during a climate of ‘limited scientific knowledge and no established treatment pathways’ (p. 1). TCF is extended by Maclean et al. positioning a preceding step to engaging with individual candidacy negotiations is to first develop notions of *collective candidacy*. Barriers to achieving this include participants facing themes of rejection, discounting and abandoning regarding LC. Reversely, facilitators for developing positive candidacy are represented by being *believed* regarding LC symptoms and illness naming, with LC patients engaging in shared involvement and responsibilities in treatment negotiations.

Although LC research utilising candidacy is sparse, similar studies to Maclean et al. exist. Liberati et al. [4] present deconstructions of a modified candidacy theory applied to understand secondary mental health service access during the C19 pandemic in England. Analysing 65 interviews, the authors highlight that restructurings in UK healthcare prioritised crisis‐focused services. This generated healthcare impacts beyond simply reduced service access. Alterations restructured service users' understandings of illness candidacy, including perceptions of *questioning* surrounding what constituted mental health issues (MHI), which MHI required attention, and patients' perceptions of deservingness and care. Factors restricted healthcare access at a time when service permeability was already reduced due to inherent healthcare pressures and restrictions from the C19 pandemic.

Taylor et al. [17] examine the experiences of 13 doctors with LC, highlighting struggles as doctors navigated healthcare systems in dual roles as providers and patients. Dubbed ‘reluctant pioneers’ (p. 833), these doctors' insider knowledge of the healthcare system revealed tensions, as they strove to understand their symptoms, and some felt ‘let down’ (p. 835) by healthcare systems. Some participants leveraged professional connections and knowledge to secure treatment, whereas others turned to alternative therapies for the first time. The challenges of balancing their dual identities as doctors and patients were highlighted, with participants eager to help others and contribute to the understanding of LC. This extended to narratives suggesting that doctors made sense of their LC experiences by framing this as ‘positive’, indicating they were better able to empathise and care for patients with unexplained symptoms. This study underscores the necessity for further research on NHS workers with LC, highlighting the importance of validating patient experiences and the unique influence of identity on illness perception and healthcare access.

## 2 Methods

This research represented a longitudinal design. Two phases of semi‐structured interviews, 6 months apart, were conducted with NHS workers experiencing LC.

### 2.1 Participant Recruitment

Participants were eligible to take part if they were employed in an NHS Scotland healthcare setting, were 18 years or older and self‐identified as having LC, with ongoing C19 symptoms for 4 weeks post‐infection [5].

### 2.2 Longitudinal Semistructured In‐Depth Interviews

Fifty participants were invited to participate in interviews (September 2021–January 2022), with second interview invitations sent 6 months later (March 2022–June 2022). Fifty interviews were completed in the first interview cycle, with 44 completed in the second cycle. Maximum variation sampling was utilised to represent a range of sociodemographic characteristics, including occupation (doctors, nurses, Allied Health Professionals [AHPs] and ancillary, admin and others [ancillary+]), as well as the severity and diversity of LC experiences and symptoms. Interviews were conducted by experienced qualitative researchers (Nick Adams, Emma MacIver and Aileen Grant), with a topic guide facilitating interviews (informed by the literature and research questions). Interviews were conducted online using MS Teams, aside from three conducted via telephone; these were digitally audio‐recorded. Data were immediately downloaded to secure university servers, then anonymised and transcribed. Table 1 shows the demographics for both interview phases.

**Table 1:** Breakdown of interviews 1 and 2 characteristics and demographic information.

	Ancillary/admin/other (ancillary+)	AHP	Medic	Nurse	Total Interviews in phase 1	Total Interviews in phase 2
BAME	1	2	2	1	6	6
Age						
≤25	0	0	0	2	2	2
26–35	1	2	1	3	7	7
36–45	4 (*−1*)^a^	3	5	1	13	12
46–55	2	3	3 (*−2*)	11 (*−1*)	19	16
56–65	3 (*−2)*	2	1	2	8	6
66+	0	0	0	1	1	1
Male	2	1	3	2	8	8
Female	8 (*−3*)	9	7 (*−2*)	18 (*−1*)	42	36
Primary care	1	1	4	6	12	12
Secondary care	9 (*−3*)	9	6 (*−2*)	14 (*−1*)	38	32
I'view 1 Total	10	10	10	20	50	
I'view 2 Total	7	10	8	19		44

Abbreviations: AHP, Allied Health Professional; BAME, Black Asian Minority Ethnic.

^a^ Bracketed numbers in italics refer to interview 2.

### 2.3 Research Context

To prevent the spread of the C19 virus, national lockdown began on 24 March 2020, with people advised to stay at home save for 1 h of daily exercise, and not mix with other households. Full stay‐at‐home restrictions were lifted on 16 March 2021 following the introduction of a tiered alert system (November 2020). All regions of Scotland were ‘unlocked’ from the tier system on 13 July 2021, but social distancing regulations remained. These enforced 1 ‐m distancing for all populated locations [18]. During this time, non‐emergency healthcare was postponed, and urgent care was reorganised around strict infection control measures.

### 2.4 Analysis

Transcripts were imported into NVivo.20 (Lumivero); analysed using a six‐stage thematic inductive approach [19, 20]. Two researchers coded and analysed raw data (Nick Adams and Emma MacIver), regularly meeting the two PIs of the research team (Aileen Grant and Nicola Torrance) and routinely the complete research team, presenting emerging findings and co‐developing themes.

Multiple data passes were completed to develop initial emergent themes. Initial code categories were generated, and themes were established. Themes were ongoingly reviewed to clarify key findings into thematic categories. In the penultimate stage, categories were re‐reviewed collaboratively, and participant data were rearranged into themes. This constructed networks of themes and subthemes representing the salient lived LC experience. Finally, clarifying phases of analysis were completed to cross‐check findings, teasing out contradictions within the established analysis. A deductive phase of analysis was conducted where initial and output thematic categories were examined for themes linked with illness candidacy.

### 2.5 PPIE/Stakeholder Workshops

During data analysis, two stakeholder workshops were conducted consisting of ‘expert advisory groups’. Individuals came from policy, clinician and research backgrounds, including some with lived experience of LC. Group discussions provided crucial insights into the real‐life challenges faced by individuals with LC, highlighting the psychological and physical toll, inconsistencies in support and the need for tailored healthcare approaches. Discussions informed data analysis, spotlighting key areas for policy improvement, such as the necessity for clearer guidelines, flexible return‐to‐work strategies and enhanced support mechanisms for LC‐affected workers. This aided in clarifying candidacy themes in the data. Sessions were held remotely via MS Teams, structured with introductions of the research team and participants, followed by breakout room discussions facilitated by two project PIs. Discussion points included the unexpected number of people still managing to work despite LC, the phased and prolonged nature of their return to work and the psychological and physical toll this takes. Participants recorded inconsistencies in support across different regions and the lack of a clear healthcare pathway for LC patients. There was a focus on the need for tailored support systems, extended phased returns and better managerial understanding and flexibility to accommodate the varied symptoms and recovery timelines associated with LC. Implications for policy highlighted the necessity for validation of LC as a legitimate condition. Recommendation ideas generated included the importance of education, flexible return‐to‐work strategies and support mechanisms to prevent the loss of valuable NHS workforce expertise.

### 2.6 Ethical Approval

Ethical approval was obtained from Robert Gordon University, School Ethical Review Panel (Reference: 21‐04). Generic review and NHS R&D approvals were obtained from all NHS boards in Scotland (IRAS: 298496). All participants gave informed consent, with verbal recorded consent at point of video and audio interviews to take part in this study.

## 3 Results

### 3.1 Factors Influencing NHS Workers' Illness Candidacy and Healthcare‐Seeking for LC

Interviews revealed the difficult reality of living with LC, with multiple, shifting symptoms impacting work and daily life. LC symptoms included debilitating fatigue, breathlessness, weakness and high heart rate, experienced cyclically and variably. Participants openly shared their experiences, distressed by LC effects. Many struggled to access healthcare, facing resistance and feeling as though little progress was being made. Understanding the need for medical help was conflicted by the C19 context, influenced by barriers to healthcare access and doubts about being taken seriously. Importance was placed upon receiving a LC diagnosis, with anxieties amplified by participants worrying about burdening the NHS and struggling to be believed regarding LC symptoms and limitations. These factors disrupted notions of illness candidacy in multiple ways.

To structure participant' narratives, new candidacy domains of *context*, *illness climate* and *sense‐making* are introduced. Context examines the impact of the pandemic healthcare context on NHS workers' candidacy. Illness climate explores low awareness of LC and reluctance to diagnose LC, affecting candidacy. Sense‐making recognises how different NHS occupational groups conceptualise LC and healthcare‐seeking based on their role identities.

### 3.2 *Context*: Healthcare in Time of C19

#### 3.2.1 Is It Really LC?

Participants across occupations and demographics described LC symptoms as severe, debilitating and ‘life‐changing’. The main obstacle hindering participants from seeking healthcare was the lack of available LC information. A prevalent theme in interviews was the doubts participants had about whether they *truly* had LC. This uncertainty (evident in both initial and follow‐up interviews) reflected participants' struggle to label and validate illness experiences, stemming from the limited information available about LC: symptoms, diagnosis and treatments. Uncertainty prevented participants from progressing beyond the illness identification stage of candidacy:

Even though I've worked in a hospital, I still feel [with supports], you weren't welcome to access them, unless you were acutely unwell. [… With the GP] you just didn't know what you were going with, maybe that was unwise, but it just didn't feel like we had the right to bother them.

(Participant 46—female nurse)

#### 3.2.2 Healthcare Access: Do I Deserve Healthcare?

During the first interviews, participants discussed delaying or attempting to manage symptoms before seeking healthcare, largely due to perceived uncertainty about their condition and perceptions of limited NHS healthcare available during the pandemic time. Follow‐up interviews revealed a growing awareness of challenges in accessing healthcare, but a recognition that things are ‘opening up again’. Despite this, participants felt overwhelmed by the influx of people already seeking care. This thinking, especially among nursing, AHP, and ancillary+ workers, led to feelings that participants were less deserving of healthcare compared to other groups. Participants believed that those with more urgent medical needs should have priority, and thus often withdrew from care themselves to increase the chances of others gaining access:

I think the system's just under so much pressure … I worry that Long COVID's just going to chucked to the backburner because acute COVID's the priority and the backlog of everything else, all the cancers and cardiac treatments and everything else that's been held up.

(Participant 37—female nurse)

#### 3.2.3 Personal Pressures and ‘Guilt’ Over Initial Help‐Seeking for LC Symptoms

Participant narratives also revealed profound guilt regarding LC illness and absence from work. Guilt frequently manifested as a barrier to seeking initial healthcare support, particularly, among nurses, AHPs and ancillary+ groups. A prevalent belief emerged: as NHS employees, participants' foremost obligation was to ‘shield’ the NHS from overburdening, leading many to refrain from seeking—or at times even openly discussing—their LC with healthcare practitioners and work colleagues. Reluctance stemmed from a desire from participants to alleviate the NHS's workload by what seemed to be a sacrificing of their own healthcare needs. Findings underscore how prevailing illness context shapes illness candidacy, as feelings of guilt and self‐deprecation impede progression towards seeking healthcare and advancing through candidacy stages:

There was lots a guilt and that because I was off work for such a long time. And it was, it was so difficult, and emotionally, I wasn't in a good place last summer ….

(Participant 6—female nurse)

### 3.3 The *Illness Climate* of LC

At the second interview, most participants had attempted to seek help from their GP; however, three participants had not. This was linked to perceptions of futility relating to ‘what could be done’ about LC symptoms, concerns over being diagnosed with LC and implications this may have over a future working in the NHS.

Over half of the participants had an LC diagnosis (28) by the second interview, although this was often presented in clinical notes as presumed or ‘probable’. Twenty‐two participants had no diagnosis of LC; their LC was not acknowledged by their GP. Between interview phases 1 and 2, participants underwent a gradual process of understanding and acknowledging their need for healthcare for LC symptoms, navigating through various stages of candidacy sense‐making and identification. Initially, they turned to online communities, akin to Maclean et al.'s [6] findings, to seek validation and understanding of LC. However, many later withdrew, shunning social media due to negative influences, particularly, as social media often portrayed detailed stories of people being let down by the NHS, which amplified guilt among participants, all of whom were NHS workers themselves.

Despite this, over time, many participants realised that their symptoms were persistent and worsening, compelling them to recognise that they needed to seek healthcare. The realisation was intertwined with disruptions caused by LC in their daily lives, including—similar to findings in Taylor et al.'s study [17]—impacts on their ability to work, engage in physical activities and maintain familial and social responsibilities. A nurse (in her first interview) articulated the impact of LC on her ability to work before being signed off sick:

… the worry for me at work [was] when I was getting things messed up on paper. Obviously, working in a hospital, you have to be careful what you're doing because if someone's got an allergy and you're writing their name on the wrong menu and they eat that, that's on your head, or even if I'm on blood stickers and I print out the wrong patient's name, you know, it can cause all sorts of problems and I said that to my boss when I ended up going off sick, … ‘look, it's actually dangerous for the patient's me being here, [it's] dangerous for me, but it's dangerous for them’ ….

(Participant 36)

As the healthcare context began to ease, participants noted a shift in their perspectives, with interviews revealing a sense of optimism as healthcare services started to open up again. This shift contributed to a reduction in the constraints of candidacy domains of questioning, deservingness and guilt, allowing participants to reconsider their healthcare‐seeking behaviours for LC. Regarding questioning, participants navigated through emerging and conflicting LC information, balancing medical definitions and prioritising rest and recovery with personal experiences shared in online communities. This process reaffirmed their newly formed *identification* of LC, empowering participants to seek help from healthcare professionals and challenge resistances encountered during revisited healthcare‐seeking. Furthermore, participants began to reframe their perceptions of deservingness and guilt. Many began to acknowledge that prioritising their own health and achieving diagnosis and treatment for LC symptoms was critical to positively move forward with their lives. This reframing process allowed participants to overcome feelings of guilt and shame that had previously prevented them from prioritising their well‐being, understanding that returning to work and contributing to the struggling and overburdened NHS necessitated being in good health. However, despite advancements in understanding and acceptance of LC, participants routinely encountered challenges in accessing healthcare, which disrupted their candidacy domains of *permeability* and *presentation*. This created a feedback loop that again placed their illness identification into question, highlighting complexities and nuances involved in the healthcare‐seeking journey and the fragility of constructing LC identification and legitimacy.

#### 3.3.1 Striving to Be Taken Seriously

In the second phase of interviews, many participants were still striving to have their LC symptoms taken seriously due to the lack of LC information available for clinicians. Perspectives were supported by the Medics sampled, speaking of their own experiences as dual‐role clinicians and patients, supporting many of the themes highlighted by Taylor et al. [17]. One female Medic—a GP—summarised this position in her second interview:

Nobody is even looking into anything [related to LC symptoms]. It's just, oh, we'll do your ECG and, you know, it's just more of the same. … COVID is new. … There's no, there's no way of looking at everything together.

(Participant 11)

Participants felt interactions with primary care professionals pushed them back into doubting the legitimacy of their symptoms. This regression in their illness identification process, termed ‘striving to be taken seriously’, was exacerbated by healthcare professionals' reluctance to diagnose LC based solely on symptoms, especially if participants had not tested positive for C19. This led participants to doubt their LC, and ‘disbelief’ angered participants, as these NHS workers mostly worked within primary and secondary care at the height of the pandemic and regularly discussing having insufficient access to usable PPE. Additionally, nurses, AHP and ancillary+ workers all discussed the unavailability of C19 tests at early pandemic stages. Participants articulated that refusing diagnosis or forward referral based on the absence of a confirmed C19 test was unfair and unjust. Others spoke of the ‘invisibility’ of symptoms, and of ‘not being believed’ in the workplace, adding to tensions, shame and guilt surrounding seeking healthcare and workplace‐based support. One participant in the ancillary+ group explained during their second interview:

[with LC] … they are all invisible symptoms. You've not fell and broke your leg or you've not had a bereavement or, you know, you've not, there's not something they can see, that's what's wrong … [they] think oh come on, you know, you're not really ill, you could go out and get a job, you're no really stressed … they're just wanting some time off their work.

(Participant 28)

#### 3.3.2 The LC Support Payment

Some participants were signed off work, receiving LC support payments actioned by the Scottish Government (13 participants in interview phase 1 were signed off and 9 participants in interview phase 2). Discussions of payments revealed links between payment eligibility and participants having a confirmed LC GP diagnosis. A small number of participants stated that they were ‘ineligible’ for LC payments despite being off sick because they were unable to attain a LC diagnosis from their GP. These participants did not have proof of an earlier positive C19 test from their initial infection. This complicated diagnoses, adding additional layers of anxiety and anger to some NHS workers, with some ‘pushing’ for an LC diagnosis to ensure they received the LC payment. One participant—a nurse—articulated this best:

… I'm struggling at the minute … being off work, to prove I had COVID away back March. Because [that] impacts the leave that I'm entitled to, at the minute I'm on sick leave, but if I could prove I had COVID I'd be on special leave, which wouldn't accrue sick leave ….

(Participant 34)

Furthermore, LC diagnoses were rarely straightforward. Some participants discussed receiving a diagnosis but marked as ‘Long COVID?’ with a question mark used to query whether this diagnosis was accurate. This complicated participants' ability to receive the government LC payment due to the ambiguous diagnosis. Also, some discussed tendencies for physical symptoms to be attributed to ‘anxiety’. Such participants were often offered anxiety and antidepressant medications instead of further investigations examining whether symptoms were LC and routed in physical as opposed to mental origins. This was interpreted as ‘downplaying’ symptom seriousness, clashing with ‘patient‐defined’ LC perspectives many found helpful when searching online. This perspective speaks to Maclean et al.'s findings [6], drawing parallels with vanguard LC patients and Pearson et al.'s [7] work prioritising LC symptoms, effects and impacts over medicalised perspectives alone. An ancillary worker articulated these tensions in their first interview, discussing the singular focus of their healthcare experience:

If it's a specific [LC] symptom … they focus on that one symptom … they don't see it as part of this whole picture of long COVID and how long it's been going on. And I just get snap decisions, you know … speaking to a doctor I've never met before, who [hasn't] had time to read the notes. I've just had a couple of minute[s] phone conversation, and that's it.

(Participant 16)

A further powerful example was provided by one nurse in her mid‐20s during her first interview. She became unwell with C19 when caring for a C19 patient, without PPE. She explained her frustrations trying to attain an LC diagnosis and support for her ongoing symptoms:

I felt really daft, like I would be going to my GP for [Long COVID symptoms like] my hair falling out, or I've constantly got issues with my bowels—or I feel sick all the time or my heart rate seems like it's racing all the time. And they were like, oh, you're just anxious, or it's, maybe it's because you're slightly overweight or maybe it's because you're depressed and all this type of stuff. Like there's never… [The] only person who said, who diagnosed me with Long COVID was a consultant at the hospital when I went.

(Participant 44)

The same nurse later recounted how she had taken many trips to GPs and met with what she articulated as ‘dismiss[al]’. She suggested that despite still struggling with LC symptoms, she would be ‘anxious’ [resisting] seeking further medical help, due to the emotional effects she experienced from not being believed. Her negative experiences of attempting *professional adjudication* caused a reappraisal of her perception of *navigation*: awareness of healthcare services available and negotiation of these, and *permeability*: the ease at which individuals access and use services. The participant explained in her second interview:

I'm not taken seriously cause, you know, … I'm young, … when I go into my GP, I don't broadcast that I'm a nurse … because I feel like I should be getting help regardless of my profession, regardless of my gender, regardless of … and I have not. … The only time I was taken seriously was when I was really unwell in the hospital bed on CPAP [Continuous Positive Airway Pressure Therapy] … But then as soon as I was out of hospital, and I went to my GP with the doctors later, they're like, you'll be fine in a couple of weeks, but just give it time.

(Participant 44)

Like the example mentioned earlier, participants commonly felt dismissed. While critical of care, some participants also reported positive experiences, discussing kindness and understanding, but balancing this with a recognition of a fundamental lack of knowledge about how they could be treated and supported to recover from LC. By phase 2 interviews, LC‐specific clinics and specialists emerged, with a small number of participants referred by their GPs, thus improving some perceptions of healthcare and developing new pathways of focus regarding symptom treatment. Findings in this theme highlight the fragility of illness candidacy despite efforts to recognise the need for LC healthcare. Negative healthcare experiences often disrupt candidacy, leading individuals to question the legitimacy of their illness and the expertise of primary care services in providing adequate support for LC. Thus, many withdraw from accessing healthcare, remaining unwell and often unable to return to NHS work.

### 3.4 *Sense‐Making* of LC Illness: A Role‐Identity Perspective

Important to our findings, Medics varied in their understanding of LC and healthcare‐seeking behaviours to other groups. Previous sections explored the impact of *healthcare context* and *illness climate* on reluctance to seek healthcare for LC. However, participants in the Medic category showed different barriers and healthcare access motivations. Medics held distinct identities that shaped their healthcare‐seeking behaviours, with understandings of LC deeply tied to a ‘medical professional identity’. Unlike other groups, Medics were certain about their symptoms being related to LC, which impacted the activation of the Candidacy domain of *professional adjudication* in unique ways for Medics (compared with other groups). Medics extensively researched LC, reading medical literature and considering various treatment options, enabling them to bypass many doubts and uncertainties faced by others. Medics self‐diagnosed based on their knowledge and experiences, swiftly engaging healthcare services for diagnosis and treatment. While other groups hesitated due to uncertainty and guilt, constraining their ability to engage in *professional adjudication*, Medics instead actively sought healthcare without questioning, viewing this as essential for their return to ‘frontline’ duties. Thus, Medics held unique dividends; medical language, skills and experiences to convince GPs that their LC was ‘real’. This lessened stresses of disbelief faced by other groups. One GP, who discussed his experiences in both interviews recognised his unique position; utilising medical knowledge to self‐advocate and secure treatments, but acknowledged other ‘non‐medical’ persons would be unable to do this:

No doctor or nurse or physiotherapist has ever tested me for POTS [Postural Tachycardia Syndrome], it's me that's tested myself and that's a huge concern … the reason I'm going for these tests … is that I've pushed for them because I've read up on what's going on and I've got better awareness of what's going on….

(Participant 32)

He elaborated on POTS in his second interview, advising he was able to persuade his GP to prescribe a combination of medications to treat this, with some success, validating his own self‐diagnosis. Despite this, Medic's LC status *was* often questioned by GPs and healthcare providers. This caused acute and unique frustrations, anger and anxieties like other groups. Importantly, and as with Taylor et al. [17], Medics struggled to reconcile their new dual identities as both receivers and providers of care. However, unlike Taylor et al., some of the Medics sampled in this research continued to work, although with reduced hours and duties. Medics disrupted identities were experienced as two interlinked motifs: guilt at being absent from work but with reduced guilt (compared with other groups) linked to healthcare‐seeking. Saliently, Medics frequently questioned their identities, explaining if they were not working as a Medic they questioned their purpose. One Medic, a female GP, highlighted this in her first interview:

I was I signed off for quite prolonged period of time, which I'm, quite frankly, mortified about. I felt really guilty because I was leaving my colleagues in the middle of a pandemic and I felt useless. … For a doctor, that's quite a lot of your identity is what you do … you feel a bit lost, because your whole identity shifts that you don't know if you're going to be able to do what you have trained and done for 20 years.

(Participant 32)

Medics' motivations for recovering from LC were two‐fold; recovery was sought, like others, to attain recovery but also to lessen the impact and burden of ongoing identity disruptions—their questioning of purpose and self‐worth. This could only be achieved by returning to a comparable state of good health and then returning to work as a Medic. Additional recovery motivations were also uncovered. GP‐partner‐Medics spoke of being ineligible to receive the LC government payment; operating as self‐employed practice partners. This additionally pressured recovery‐seeking, Medics discussing partner pressures to return to work or resign, and only receiving full pay for 1 year before this would be reduced or stopped completely. This added significant stresses to Medics affected, making recovery a priority for both identity, health and financial reasons.

### 3.5 Discussion: LC: A Case for Disrupted Candidacy

Our research reveals that participants' abilities to develop candidacy were disrupted in unique ways by LC. This occurred via wider LC *context* and *climate* influencing the *operating conditions* within which illness candidacy could be established—constraining all participants from developing *identification* surrounding LC symptoms. However, blocked operating conditions were negotiated by participants. Negotiation was linked to factors identified in Maclean et al. [6]. Interviews in this study revealed increases in participants' confidence and authority regarding the ‘patient defined’ nature of LC. This represented (as with Maclean) a collection of diverse and severe symptoms, solidified by accounts in online support groups validating experiences and constructing illness legitimation. This operated alongside reductions in guilt at seeking healthcare services, underpinned by realisations the ongoing nature and severity of participants' LC dictated them as deserving of healthcare. This was combined with the NHS ‘opening up’. However, for most nurses, AHPs and ancillary+ workers, engagements at the *professional adjudication* and *offer or rejection of services* stages rapidly destabilised their established illness candidacy and subsequently placed the *identification, navigation, permeability, presentation* domains under threat, with many participants regressing—questioning whether their LC illness was ‘real’ and their deservingness of diagnosis and treatment. These participants felt available healthcare was neither LC‐focused nor capable of adequately diagnosing and treating LC. Thus, participants' forward candidacy journeys were disrupted with the activation of later candidacy domains constructing a negative feedback loop that saw participants returning to and then stuck at an early stage of trying to identify themselves as requiring healthcare. This process differed somewhat for the Medics. As mentioned earlier, Medics readily *adjudicated* for themselves; however, they suffered from unique identity disruptions and struggled to reconciling their provider/patient status, experiencing unique guilt and frustration. This triggered blocking at the *operating conditions* candidacy stage that impacted *navigation* and service availability/acceptance in similar ways to others.

**Figure 2:**
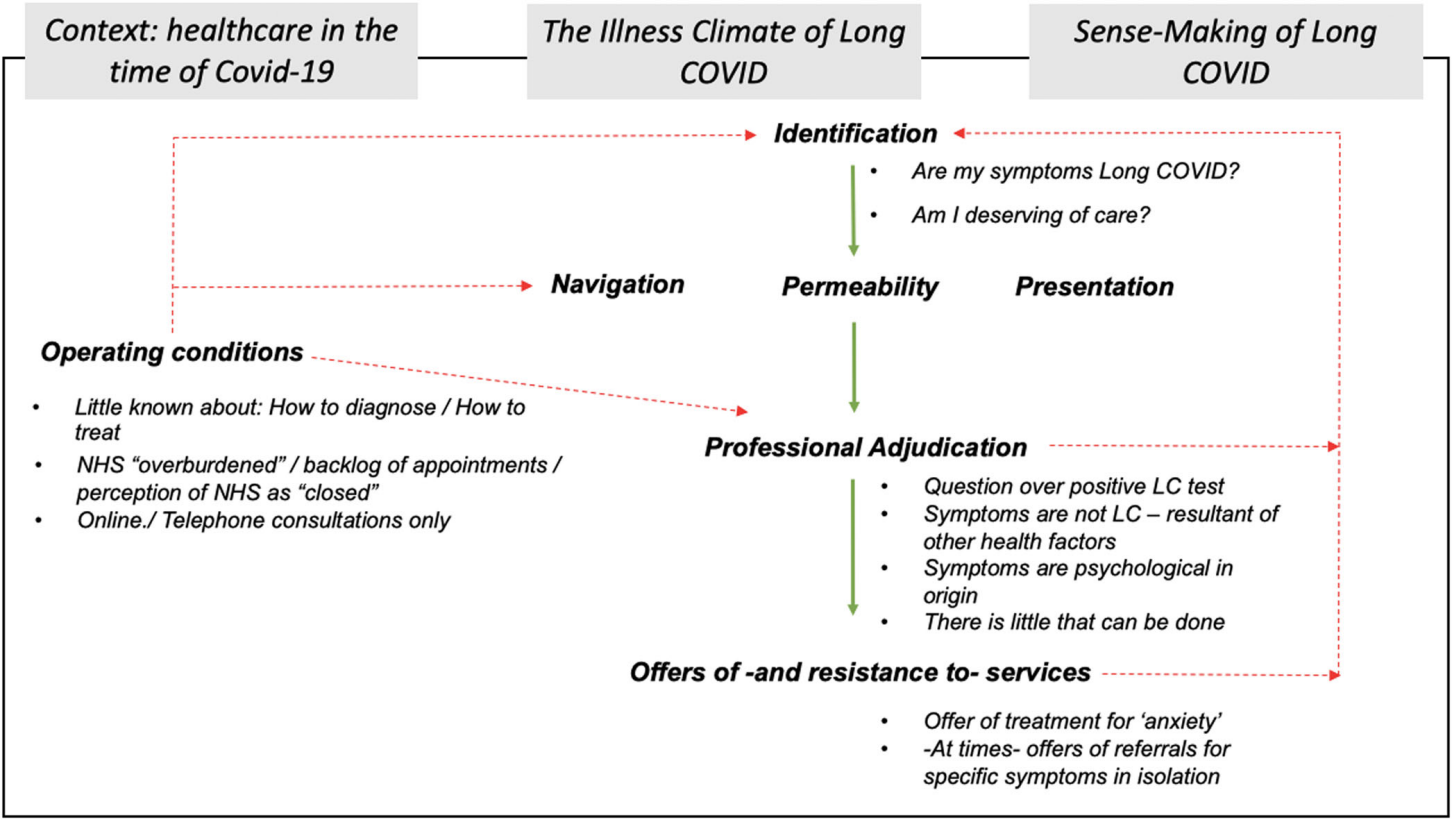
Diagram showing a possible linear pathway of progression for NHS workers with LC seeking healthcare for their symptoms (shown by downward green arrows). Broken lines in red highlight the disruptions to this pathway, recurrently pushing NHS workers back to the initial Identification stage of candidacy negotiations. The newly introduced ordering domains reveal and anchor participant's candidacy experiences and are shown in grey boxes, atop the framework. Explanatory text linking specific influences of these domains over established candidacy domains is shown by bullet points.

The participants' lived experiences are demonstrated in Figure 2, which presents a reworked candidacy model depicting the constrained healthcare access journeys of NHS workers in Scotland seeking primary care support for LC illness.

Several important considerations are evident when considering unique factors that constrain NHS workers from seeking and receiving healthcare for LC symptoms. Saliently, TCF is—at times—presented as a linear pathway of progress to identifying illness, need for treatment and seeking healthcare [13]. Although TCF (Figure 1) outlines *operating conditions* as an overarching influence over domains, the pathway of progression through the framework remains continuous, beginning with *identification*, before moving to navigation, *permeability, presentation, professional adjudication* and *offer or rejection of services*. Others have discussed issues with this linearity, applying TCF in a non‐linear structure to allow theory to fit flush with the lived experience of human actors and their complex illness and health‐seeking journeys [16, 21, 22].

This research demonstrates that the most important negative effects over *identification* of LC illness and a need to seek healthcare were constructed from participants experiencing a lack of being believed with regard to LC symptoms, a lack of having LC symptoms legitimised through diagnosis, occurring at the *professional adjudication* stage. Findings regarding disbelief support some existing literature identifying the impacts of ‘medical gaslighting’ [8]. As shown in Figure 2, a negative feedback loop of influence disrupts participants' candidacy, returning them to the beginning initial *identification* stage of the framework and preventing progressing beyond this. This paradigm ultimately resulted in some participants withdrawing from help‐seeking for LC, caught in a loop of questioning the ‘realness’ and legitimacy of LC, while also, in some cases being both unable to work and to live a full, functional and fulfilling life.

The implications for NHS workers with LC underscore the fragility of illness candidacy, shaped by complex journeys of negotiation and sense‐making. Importantly, *being believed* regarding LC symptoms is pivotal for encouraging healthcare‐seeking and continued engagements with healthcare professionals. This suggests that occupational support should prioritise fostering *belief* to facilitate return‐to‐work and well‐being for NHS staff currently struggling with return‐to‐work processes while experiencing LC. Near universally, NHS workers expressed a strong desire to return to work despite ongoing symptoms, suggesting a need for supportive return‐to‐work initiatives and adjustments. Theoretical implications highlight the limitations of linear frameworks like TCF in capturing the complex and non‐linear nature of healthcare‐seeking journeys. Instead, TCF may be reconceptualised as a constraint‐based framework (Figure 2), illuminating barriers to healthcare access and guiding future research and interventions to promote rapid healthcare access for complex illnesses like LC.

## 4 Conclusion

This study provides a longitudinal view of NHS workers grappling with accessing healthcare for LC symptoms. By applying an adapted illness candidacy theory to NHS workers in Scotland with LC, our approach uncovers complex barriers to seeking healthcare. Our study integrates three key dimensions related to the C19 pandemic into the framework: the pandemic healthcare context, low GP knowledge about LC and the influence of participants' role identities. The application reveals ‘*Disrupted Candidacy*’, an explanatory framework where NHS workers face intersecting challenges hindering their ability to seek and receive LC healthcare. Research highlights the arduous process of developing illness candidacy, marked by doubt, uncertainty and effort. Even when LC identification is achieved, this is often rapidly undermined by factors such as low GP knowledge and unclear diagnosis and treathment pathways. Findings shed light on why NHS workers resist healthcare‐seeking and the complexities of navigating healthcare access and its impact on illness identification. This study offers evidence‐based suggestions for workplace support and proposes a modified version of TCF for examining healthcare access in the context of complex illnesses like LC. This approach may help identify and visualise barriers to healthcare access, encouraging a deeper understanding of healthcare‐seeking challenges. Further research may use the *Disrupted Candidacy Framework* to reveal the complex interplay between the activation of different domains of candidacy invalidating, truncating or negatively influencing other domains. This can enhance understanding of the hidden struggles regarding illness help‐seeking, particularly the undeniable impact of overarching domains such as the local healthcare context and illness climate and how personal sense‐making of illness is constructed and maintained by these two factors.

## Author Contributions

**Nicholas Norman Adams:** writing (lead)–original draft, conceptualisation (lead), formal analysis, investigation. **Emma MacIver:** review and editing, conceptualisation support, formal analysis, investigation. **Flora Douglas:** review and editing, conceptualisation support. **Catriona Kennedy:** review and editing, conceptualisation support. **Diane Skåtun:** conceptualisation support, funding acquisition support. **Virginia Hernandez Santiago:** conceptualisation support, funding acquisition support. **Nicola Torrance:** review and editing, funding acquisition (lead), conceptualisation support, formal analysis support. **Aileen Grant:** review and editing, funding acquisition (lead), conceptualisation (lead), formal analysis support, investigation, supervision (lead).

## Ethics Statement

Ethical approval was obtained from Robert Gordon University, School Ethical Review Panel (Reference: 21‐04). Generic review and NHS R&D approvals were obtained from all NHS boards in Scotland (IRAS: 298496). All participants gave informed consent, including verbal recorded consent to take part in this study.

## Conflicts of Interest

The authors declare no conflicts of interest.

## Data Availability

Data are not available but are stored securely and anonymously.
